# Low one‐repetition‐maximum knee extension is significantly associated with poor grip strength, female sex, and various aging‐related syndromes

**DOI:** 10.1002/agm2.12109

**Published:** 2020-04-28

**Authors:** Sunny Singhal, Rishav Bansal, Gevesh Chand Dewangan, Ashish Datt Upadhyay, Sada Nand Dwivedi, Prashun Chatterjee, Avinash Chakrawarty, Aparajit Ballav Dey

**Affiliations:** ^1^ Department of Geriatric Medicine All India Institute of Medical Sciences New Delhi India; ^2^ Department of Biostatistics All India Institute of Medical Sciences New Delhi India

**Keywords:** one repetition maximum, geriatric syndromes, knee extension strength, lower limb strength

## Abstract

**Objective:**

Muscle strength in older adults is usually measured according to grip strength, which demonstrates upper muscle strength only. In this study, we used one‐repetition‐maximum (1‐RM) knee extension as a measure of lower limb strength and assessed its relationship with grip strength and various geriatric syndromes.

**Methods:**

One hundred outpatients over the age of 65 years were recruited from a geriatric medicine center in India. The 1‐RM knee extension was measured along with grip strength. Various geriatric conditions were measured, such as: nutrition (using the Mini Nutritional Assessment), cognition (Hindi Mental State Questionnaire), depression (5‐item Geriatric Depression Scale), frailty (Fried and Rockwood models), and osteoporosis (dual‐energy X‐ray absorptiometry scan). Sarcopenia was diagnosed using the Asian Working Group for Sarcopenia criteria.

**Results:**

The mean age of participants was 72.5 years with 69% of them male. Median values of 1‐RM knee extension and grip strength were 2.29 (0.5‐10.0) and 17.5 (0‐78), respectively. The 1‐RM knee extension had moderate correlation with grip strength (*r* = 0.491, *P* < 0.001). Among demographic details, only female sex (*P* < 0.001) was significantly associated with lower 1‐RM values. Further, after adjusting for age and sex, lower value of log_10_ 1‐RM knee extension was found to be significantly associated with malnutrition (*P* = 0.001), dementia (*P* = 0.016), depression (*P* = 0.047), frailty (Rockwood:* P* = 0.049; Fried:* P* = 0.011), and sarcopenia (*P* < 0.001).

**Conclusion:**

The 1‐RM knee extension has only moderate correlation with grip strength. A lower 1‐RM knee extension value is significantly associated with female sex and various geriatric conditions, such as malnutrition, dementia, depression, frailty, and sarcopenia.

## 1 INTRODUCTION

Good muscle strength is an important prerequisite for both basic and instrumental activities of daily living. However, aging is associated with a progressive decline in muscle strength leading to increasing functional dependence in older adults.^1^ It is also associated with various adverse health outcomes, including mortality.^2^

The ability of the muscle to generate force can be measured in several ways. Strength can be measured statically (ie, muscle contraction without any change in muscle strength) or dynamically (ie, muscle contraction with lengthening/shortening of muscle strength). Static or isometric strength (eg, grip strength) is specific to the muscle group and joint angle that is being tested and thus has a limitation in describing overall muscular strength. However, the ease of measurement makes it convenient. Dynamic or isokinetic strength (eg, one‐repetition maximum [1‐RM]) is a much better reflection of muscle function in everyday activity; however, it is difficult to measure and requires special measurement tools.^3^

The most often used and validated tool to measure muscle strength in geriatrics is maximum handgrip strength. Grip strength has been shown as a marker of functional status of older adults.^4^ Lower handgrip strength has shown to be associated with and predictive of falls, disability, increased hospital stay, and increased mortality.^5^ It has also shown association with cognitive impairment, depression, and sleep duration in older adults.^6^, ^7^, ^8^ Both the European and Asian Working Groups on Sarcopenia have recommended handgrip strength for the measurement of muscle strength while assessing sarcopenia in older adults.^9^, ^10^

However, lower limbs are more relevant than upper limbs for gait and physical function in older adults. Lower extremity strength and power are important prerequisites for poor performance in functional mobility, hence it is important to evaluate them correctly. In fact, a large proportion of functional performance activities used for assessment in the clinic include use of the lower extremities. Examples of these activities include walking, squatting, and going up and down stairs. Studies have shown that poor lower extremity performance even in the absence of disability is predictive of increased hospitalization^11^ and development of subsequent disability.^12^ Lower extremity muscle mass and strength are independent predictors of the severity of mobility disability in older adults with compromised physical functioning. There is a strong interrelationship between lower extremity muscle mass and muscle strength, and this association was extended among a group of mobility‐impaired elders who exhibited performance‐based limitations in physical functioning.^13^ However, one of the main difficulties in evaluating lower limb muscle strength is absence of valid norms for these tests.

Recently, the chair‐stand test has been recommended for testing muscle strength; however, it is only a proxy measure of quadriceps muscle strength.^9^ Further, studies have shown that the chair‐stand test time does not always correlate well with knee extensor strength^14^, ^15^ and is dependent on a number of other factors (eg, balance, and sensorimotor and psychological factors) besides muscle strength.^16^ Hence, we need a better tool for accurate measurement of lower limb strength.

The 1‐RM is defined as the maximum weight that can be lifted throughout the full movement. However, it is not always desirable for older adults to lift the maximum weight as they might have some medical restrictions. Hence, various validated prediction equations are used to predict 1‐RM based on submaximal performances.^17^ The 1‐RM knee extension test has shown to be a valid and reliable means to assess leg strength as compared with isometric and isokinetic dynamometry independent of age and/or sex.^18^, ^19^ However, its use in clinical geriatrics as a day‐to‐day tool remains to be seen as we need to see how well it performs against the existing gold standard (ie, handgrip strength) and understand other factors associated with it.

We designed this study to evaluate the utility of the 1‐RM knee extension in older adults by comparing it with handgrip strength and other demographic and clinical factors.

## 2 METHODS

This was a cross‐sectional observational study conducted in participants aged 65 years and over in the outpatient department of Geriatric Medicine, All India Institute of Medical Sciences, New Delhi during the period from July to October 2017. Participants who were suffering from critical illness or unable to undergo the detailed assessment were excluded. As there were no similar studies on this topic, a convenient sample size of 100 was chosen. Written informed consent was obtained from each subject in accordance with study protocols approved by the Institute Ethics Committee.

**FIGURE 1:**
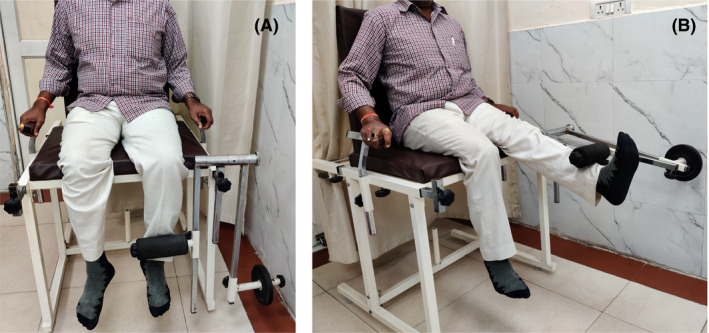
(A) Resting position and (B) extended knee position of leg while measuring one‐repetition‐maximum knee extension

To measure the 1‐RM knee extension, the subject was first asked to sit comfortably on a standard quadriceps chair with his/her back straight and against the backrest and legs hanging freely (Figure 1A). The subject was then asked to lift the shin foam roller with one leg at a time initially without any weight on the resistance arm. The range of knee joint was noted for each side. Next, weights were incrementally added to the resistance arm of one side at a time starting at 1 kg. The subject was then asked to lift the roller as many times as possible and was instructed that the motion (flexion and extension of knee) should not be jerky. The subject was asked to lift the roller to the maximum of his/her knee extension's range of motion (Figure 1B). If the subject was able to lift the roller more than 10 times, the weight was increased by 1 kg and the subject was asked to lift the weight again. The number of times the patient was able to lift the roller to his/her full range was noted along with the weight on the resistance arm. The 1‐RM knee extension of each side was calculated by using the Brzycki formula. The best 1‐RM of the two sides was recorded as the subject’s 1‐RM.

Various geriatric conditions were then assessed using standardized questionnaires. Nutrition was assessed using the Mini Nutritional Assessment. Cognition was measured using the Hindi Mental State Questionnaire in which dementia is defined by a score of <23. Depression was assessed using the 5‐item Geriatric Depression Scale in which a score ≥2 is considered abnormal. Frailty was assessed using the Fried Frailty Phenotype model and the Rockwood deficit accumulation model (Appendix S1). Osteoporosis was assessed according to the World Health Organization’s definition using a dual energy X‐ray absorptiometry scan at L1‐L4 vertebra and left neck of femur. Sarcopenia was diagnosed using the criteria of the Asian Working Group for Sarcopenia (AWGS). Grip strength was assessed using a hand‐held dynamometer using standardized protocol.^20^

Statistical analysis was done using STATA V. 14. Descriptive statistics, including absolute frequency distribution, percentage distribution, mean, median, range, and standard deviation, were calculated as appropriate. The 1‐RM parameter was assessed for normal distribution using the Shapiro‐Wilk test and log_10_ transformation of 1‐RM value was carried out to achieve normality assumption. The unpaired *t* test was used to compare log_10_ 1‐RM between two groups. Similarly, if more than two groups were to be compared, one‐way analysis of variance (ANOVA) followed by post hoc comparison with Bonferroni correction was done. Analysis of covariance was carried out for differences among groups in log_10_ 1‐RM value after adjusting for age and sex. To determine the strength of the relation between grip strength and 1‐RM, Spearman’s correlation coefficient was used. Statistical significance was set at a *P* value < 0.05.

## 3 RESULTS

Table 1 shows the baseline characteristics of the 100 participants included in the study. The mean age of the participants was 72.5 years with mostly male predominance (69%). Median 1‐RM knee extension was 2.29 kg (0.5‐10 kg) and median grip strength was 17.5 kg (0‐78 kg). Forty‐eight percent of subjects were either at risk or had poor nutritional status and 82% had low bone mineral density (osteopenia or osteoporosis). Seventeen percent and 40% of the participants were also screened to have dementia and depression, respectively. A significant proportion of the population was diagnosed as frail either according to the Rockwood (44%) or Fried (42%) models. Further, 53% of the participants were diagnosed as having sarcopenia according to the AWGS criteria.

**TABLE 1:** Basic characteristics and geriatric conditions of the study population (n = 100)

Serial number	Variables	Measure
1	Age^a^	72.5 ± 6.4 y
	65‐74 y (%)	69 (69)
	≥75 y (%)	31 (31)
2	Sex	
	Male	69 (69)
	Female	31 (31)
3	Body mass index (kg/m^2^)^a^	23.62 ± 4.38
	<18.5	9 (9)
	18.5‐22.9	33 (33)
	23.0‐24.9	25 (25)
	25.0‐29.9	25 (25)
	≥30.0	8 (8)
4	Grip strength (kg)^b^	17.5 (0‐78)
5	1‐RM knee extension (kg)^c^	
	Mean ± SD	2.71 ± 1.61
	Median (range)	2.29 (0.5‐10.0)
6	Nutrition (Mini Nutritional Assessment)	
	Normal	52 (52)
	At risk	35 (35)
	Malnourished	13 (13)
7	Osteoporosis	
	Normal	18 (18)
	Osteopenia	44 (44)
	Osteoporosis	38 (38)
8	Dementia	
	Yes	17 (17)
	No	83 (83)
9	Depression	
	Yes	40 (40)
	No	60 (60)
10	Rockwood Frailty Index	
	Frail	44 (44)
	Non‐frail	56 (56)
11	Fried Frailty Phenotype	
	Frail	42 (42)
	Non‐frail	58 (58)
12	Sarcopenia	
	Yes	53 (53)
	No	47 (47)

Notes

All other variables are presented as n (%).

Abbreviation: 1‐RM, one‐repetition maximum.

^a^ Age and body mass index are presented as mean ± SD.

^b^ Grip strength is presented as median (range).

^c^ The 1‐RM knee extension is presented as both mean ± SD and median (range).

The 1‐RM knee extension showed moderate but statistically significant correlation with grip strength (*r* = 0.491, *P* < 0.001) and was further significantly associated with sex but not with age or body mass index (BMI; Table 2). After adjusting for age and sex, the log_10_ 1‐RM knee extension was found to have a statistically significant association with nutrition status, dementia, depression, frailty, and sarcopenia but not with osteoporosis (Table 3).

**TABLE 2:** Association of 1‐RM knee extension with baseline conditions (n = 100)

Serial number	Variables (n)	1‐RM knee extension (Mean ± SD)		*P* value
		Actual value	Log_10_ value	
1	Sex^a^			
	Female (31)	1.94 ± 0.89	0.24 ± 0.21	**<0.001**
	Male (69)	3.06 ± 1.74	0.42 ± 0.25	
2	Age^a^			
	65‐74 (69)	2.83 ± 1.68	0.38 ± 0.24	0.194
	≥75 (31)	2.44 ± 1.40	0.31 ± 0.27	
3	Body mass index^b^			
	<18.5 (9)	2.46 ± 2.74	0.26 ± 0.31	0.126
	18.5‐23.0 (33)	2.36 ± 1.06	0.32 ± 0.24	
	23.1‐25.0 (25)	3.29 ± 1.80	0.45 ± 0.25	
	25.1‐30.0 (25)	2.91 ± 1.50	0.40 ± 0.24	
	>30.0 (8)	2.17 ± 1.05	0.28 ± 0.24	

The significant *P*‐values (<0.05) have been highlighted in bold.

Abbreviation: 1‐RM, one‐repetition maximum.

^a^ *t* test.

^b^ One‐way ANOVA with post hoc comparison using Bonferroni test.

**TABLE 3:** Association of log_10_ 1‐RM knee extension with geriatric conditions (n = 100)

Serial number	Variables (n)	1‐RM knee extension (Mean ± SD)		*P* value	
		Actual value	Log_10_ value	Unadjusted	Adjusted^a^
1	Nutrition^b^				
	Normal^1^ (52)	2.89 ± 1.47	0.41 ± 0.22	**0.001** **1 versus 3—0.001** **2 versus 3—0.035**	**0.001** **1 versus 3—0.001** **2 versus 3—0.005**
	At risk^2^ (35)	2.36 ± 1.12	0.33 ± 0.20		
	Malnourished^3^ (13)	1.48 ± 0.77	0.13 ± 0.19		
2	Osteoporosis^b^				
	Normal^1^ (18)	3.43 ± 1.78	0.48 ± 0.24	**0.003** **1 versus 3 = 0.007** **2 versus 3 = 0.023**	0.094
	Osteopenia^2^ (44)	2.9 ± 1.43	0.41 ± 0.23		
	Osteoporosis^3 ^(38)	2.17 ± 1.58	0.26 ± 0.25		
3	Dementia^c^				
	No (83)	2.9 ± 1.64	0.40 ± 0.24	**0.012**	**0.016**
	Yes (17)	1.81 ± 1.07	0.18 ± 0.27		
4	Depression^c^				
	No (60)	2.94 ± 1.70	0.41 ± 0.23	**0.033**	**0.047**
	Yes (40)	2.37 ± 1.40	0.30 ± 0.27		
5	Rockwood Frailty Index^c^				
	Non‐frail (56)	3.04 ± 1.76	0.42 ± 0.24	**0.012**	**0.049**
	Frail (44)	2.31 ± 1.31	0.29 ± 0.26		
6	Fried Frailty Phenotype^c^				
	Non‐frail (58)	3.07 ± 1.70	0.43 ± 0.23	**0.001**	**0.011**
	Frail (42)	2.22 ± 1.34	0.27 ± 0.26		
7	Sarcopenia^c^				
	No (47)	3.38 ± 1.82	0.47 ± 0.23	**<0.001**	**<0.001**
	Yes (53)	2.12 ± 1.11	0.27 ± 0.23		

Since nutrition and osteoporosis have more than 2 components, a superscript (1, 2 & 3) is given for each component so that *P*‐values between individual components can be correctly defined.

The significant *P*‐values (<0.05) have been highlighted in bold.

Abbreviation: 1‐RM, one‐repetition maximum.

^a^ Adjusted for age and sex ANCOVA applied.

^b^ One‐way ANOVA with post hoc comparison using Bonferroni test.

^c^ *t* test.

## 4 DISCUSSION

This study was aimed at understanding the utilization of the 1‐RM knee extension as a marker of lower limb muscle strength in clinical practice and its correlation with upper limb muscle strength measure (ie, grip strength). In this study, 1‐RM knee extension and grip strength were moderately correlated. So, while upper limb and lower limb muscle strength are correlated with each other, the correlation is only moderate and hence lower limb muscle strength needs to be assessed separately. Bohannon et al^21^ reported that dynamometer measurements of the upper limb (grip strength) and lower limb (knee extension) can be used to characterize the strength of only those limbs from which they are obtained rather than overall muscle strength. Similarly, poor to moderate correlation has been seen between dynamometer‐measured grip strength and knee extension strength in older adults.^22^, ^23^ Hence, it is essential to use a separate measure of lower limb strength in older adults rather than using only grip strength.

Lower 1‐RM values were found to be associated with female sex but not with older age group (≥75 years). Lemmer et al showed that the changes in 1‐RM strength after a training program varied from muscle to muscle and were differentially affected by age and sex. For example, they found that while knee extension was affected by sex, knee flexion was affected by age only.^24^ Similarly, though lower values were seen in both undernourished and obese subjects, 1‐RM knee extension was found to have no significant relationship with BMI. This is similar to grip strength, which also shows no association with BMI.^25^ However, as expected, malnutrition was significantly associated with poor 1‐RM values. Adequate dietary intake, especially protein supplementation, is considered to be the most important component of any strategy to prevent loss of muscle strength.^26^

The 1‐RM knee extension was also found to be associated with dementia and depression. Various studies have shown associations between declining grip strength and increased risk of dementia^27^, ^28^ and hence grip strength is also suggested as a measure to monitor cognitive decline in older adults.^29^ Similarly, poor grip strength has shown a bidirectional association with depression in longitudinal studies.^30^, ^31^ Muscle strength, especially grip strength, has shown significant association with osteoporosis^32^ and has even been suggested as one of the risk factors for osteoporosis.^33^ Though our study initially showed a similar relationship between 1‐RM knee extension and osteoporosis, the association was statistically insignificant after adjusting for age and sex.

Poor lower limb muscle strength as measured by 1‐RM knee extension was also found to be significantly associated with sarcopenia and frailty. Poor muscle strength is considered a key component and measure of physical frailty and sarcopenia. Physical frailty is defined as a medical syndrome that is characterized by diminished strength and endurance, and reduced physiologic function that increases an individual’s vulnerability for developing increased dependency and/or death.^34^ There is no single consensus on the diagnostic criteria of frailty and there are various instruments used for frailty’s measurement. The Fried Frailty Phenotype model, which consists of five components, including poor handgrip strength,^35^ is the instrument most extensively used in frailty research and clinical studies.^36^ Though the 1‐RM knee extension has not been used in assessment or diagnosis of frailty, it has been used as an intervention in frailty with significant benefit.^37^

The initial consensus diagnostic criteria for sarcopenia, which were first developed by the European Working Group for Sarcopenia and later adopted by the AWGS, defined sarcopenia as low muscle mass with either low muscle strength or physical performance.^10^, ^38^ However, it is now recognized that poor muscle strength rather than poor muscle mass is the principal determinant of muscle failure and is a better predictor of adverse outcomes.^39^ Hence, the European Working Group updated its diagnostic criteria of sarcopenia to include muscle strength as the primary parameter in the definition of sarcopenia.

While grip strength has been defined as a measure of upper limb muscle strength, there is no direct measurement tool for lower limb muscle strength. As we report in this study, the upper limb and lower limb strength are only moderately correlated, and it is possible for individuals to have relatively good upper limb strength while having poor lower limb strength. These individuals, though being sarcopenic or frail, will miss the diagnosis because of good grip strength despite being at risk of poor functional outcomes. Hence, we propose that the 1‐RM knee extension can be used as a lower limb muscle strength measure in the diagnosis of both sarcopenia and frailty. However, it suffers the same drawback as dual energy X‐ray absorptiometry for muscle mass as the quadriceps chair is not very portable, thus limiting its role in community screening.

Though the sample size was taken based on convenience, it is still the largest study in terms of sample size that has measured 1‐RM in older adults. Earlier studies have measured and validated 1‐RM in a relatively smaller sample size.^18^, ^19^, ^40^, ^41^ Additionally, the study included all older adults irrespective of comorbidities giving a better and comprehensive representation of the older population. However, as it was a cross‐sectional study, we cannot establish a causation between two variables. Further, it was an outpatient‐based study. A larger community‐based longitudinal study is needed to further validate the results and arrive at a proper cut‐off to be used for diagnostic purposes.

In conclusion, the 1‐RM knee extension is a good tool for measurement of lower limb muscle strength in older adults. Although its correlation with handgrip strength is only moderate, it has a significant association with various geriatric conditions (sarcopenia, frailty, malnutrition, dementia, and depression). The 1‐RM knee extension should be used as an independent tool to measure lower limb muscle strength in both research and clinical practice.

## CONFLICTS OF INTEREST

Nothing to disclose.

## AUTHOR CONTRIBUTIONS

All authors: Writing of paper. Sunny Singhal: Design, data collection, statistical analysis. Rishav Bansal: Data collection, statistical analysis. Gevesh Chand Dewangan: Data collection. Ashish Datt Upadhyay: Statistical analysis. Sada Nand Dwivedi: Design, statistical analysis. Prashun Chatterjee: Design, data collection. Avinash Chakrawarty: Design, data collection, literature review. Aparajit Ballav Dey: Design, literature review, statistical analysis, coordination.

## Supporting information

Appendix S1
