# Supplementary material for: Low one‐repetition‐maximum knee extension is significantly associated with poor grip strength, female sex, and various aging‐related syndromes
Source: Aging Med (Milton). 2020 Apr 28;3(2):125–31. doi: 10.1002/agm2.12109 (PMC7344850; doi:10.1002/agm2.12109)
Supplement: Supplementary file 1 — Appendix S1 [file AGM2-3-125-s001.docx]

Supplement 1: Fried phenotypic model of frailty^1^

Frailty was defined if 3 or more variables were present:

| i. | Weight loss | >4.5 kg loss in 1 year |
| --- | --- | --- |
| ii. | Walking speed* | Time to walk distance of 4 meter: |
| iii. | Grip strength** | Right: Left: Avg: |
| iv. | Exhaustion: | The CES-D 2 questions are:   1. “I felt that everything I do is an effort”. 2. “I cannot get going.”   Response of a moderate amount of time (3-4 days) or most of the time to either of the question is positive |
| v. | Low physical activity | Fried used Minnesota Leisure Time Activity Questionnaire short version for measurement of physical activity. However, since it is not applicable in Indian settings, we used a simpler question as used in the simplified Women’s Health Initiative study^2^.  How often do you walk outside the home for more than 10 min without stopping?  Response of less than 2–3 times each week is considered as positive |

* Positive if 4 m walking speed time is above the gender and height stratified cut-off time

| **Height** | **Cut-off time for walking speed** |
| --- | --- |
| Men | |
| ≤173 cm | ≥ 7 seconds |
| >173 cm | ≥ 6 seconds |
| Women | |
| ≤159 cm | ≥ 7 seconds |
| >159 cm | ≥ 6 seconds |

** Positive if average grip strength is below gender and BMI stratified cut-off values

| **BMI** | **Grip strength** |
| --- | --- |
| Men |  |
| ≤24 | ≤29 |
| 24.1-26 | ≤30 |
| 26.1-28 | ≤30 |
| >28 | ≤32 |
| Women |  |
| ≤23 | ≤17 |
| 23.1-26 | ≤17.3 |
| 26.1-29 | ≤18 |
| >29 | ≤21 |

Supplement 2^3^:

Rockwood Frailty index: The 36 variables used for calculating Rockwood frailty index are mentioned below. Subjects with nine or more deficits (Frailty index ≥0.25) were considered as frail.

1) Has long-term disability or handicaps

2) Restriction of activities

3) Needs help for preparing meals

4) Needs help for shopping for necessities

5) Needs help for house work

6) Needs help for heavy household chores

7) Needs help for personal care

8) Needs help moving about inside house

9) Has arthritis or rheumatism

10) Has high blood pressure

11) Has chronic bronchitis or emphysema

12) Has diabetes mellitus

13) Has heart disease

14) Has cancer

15) Has stomach or intestinal ulcers

16) Suffers from the effect of stroke

17) Suffers from urinary incontinence

18) Has migraine headache

19) Has cataracts

20) Has glaucoma

21) Has other medical conditions

22) Have no regular physical exercise

23) Has vision problem

24) Has hearing problem

25) Feeling hopeless

26) Has dexterity problem

27) Has emotional problem

28) Has memory problem

29) Has bodily pain

30) Has speech problem

31) Taking 5 or more medications

32) Has difficulty carrying or lifting light loads

33) Mobility problem

34) Has limited kind or amount of activity

35) Feels tired all the time

36) Weight loss

**References:**

1. Fried LP, Tangen CM, Walston J, et al. Frailty in older adults: evidence for a phenotype. *J Gerontol A Biol Sci Med Sci*. 2001;56(3):M146-156.

2. Zaslavsky O, Zelber-Sagi S, LaCroix AZ, et al. Comparison of the Simplified sWHI and the Standard CHS Frailty Phenotypes for Prediction of Mortality, Incident Falls, and Hip Fractures in Older Women. *J Gerontol Ser A*. 2017;72(10):1394-1400. doi:10.1093/gerona/glx080

3. Song X, Mitnitski A, Rockwood K. Prevalence and 10-Year Outcomes of Frailty in Older Adults in Relation to Deficit Accumulation: FRAILTY PREVALENCE AND OUTCOME. *J Am Geriatr Soc*. 2010;58(4):681-687. doi:10.1111/j.1532-5415.2010.02764.x
